# cDNA targets improve whole blood gene expression profiling and enhance detection of pharmocodynamic biomarkers: a quantitative platform analysis

**DOI:** 10.1186/1479-5876-8-87

**Published:** 2010-09-25

**Authors:** Mark L Parrish, Chris Wright, Yarek Rivers, David Argilla, Heather Collins, Brendan Leeson, Andrey Loboda, Michael Nebozhyn, Matthew J Marton, Serguei Lejnine

**Affiliations:** 1 Covance Genomics Laboratory, LLC, 401 Terry Ave, Seattle, WA 98109, USA; 2 Clinical Development Lab, Merck & Co., Inc., 126 E. Lincoln Ave, Rahway, NJ 07065, USA; 3 Preclinical Immunology, Infectious Disease Research Institute, 1124 Columbia Street, Suite 400, Seattle, WA 98104, USA; 4 Seattle Biomed, 307 Westlake Avenue N, Suite 500, Seattle, WA 98109, USA; 5 Department of Molecular Profiling Research Informatics, Merck & Co., Inc., 33 Avenue Louis Pasteur, Boston, MA 02115, USA

## Abstract

**Background:**

Genome-wide gene expression profiling of whole blood is an attractive method for discovery of biomarkers due to its non-invasiveness, simple clinical site processing and rich biological content. Except for a few successes, this technology has not yet matured enough to reach its full potential of identifying biomarkers useful for clinical prognostic and diagnostic applications or in monitoring patient response to therapeutic intervention. A variety of technical problems have hampered efforts to utilize this technology for identification of biomarkers. One significant hurdle has been the high and variable concentrations of globin transcripts in whole blood total RNA potentially resulting in non-specific probe binding and high background. In this study, we investigated and quantified the power of three whole blood profiling approaches to detect meaningful biological expression patterns.

**Methods:**

To compare and quantify the impact of different mitigation technologies, we used a globin transcript spike-in strategy to synthetically generate a globin-induced signature and then mitigate it with the three different technologies. Biological differences, in globin transcript spiked samples, were modeled by supplementing with either 1% of liver or 1% brain total RNA. In order to demonstrate the biological utility of a robust globin artifact mitigation strategy in biomarker discovery, we treated whole blood *ex vivo *with suberoylanilide hydroxamic acid (SAHA) and compared the overlap between the obtained signatures and signatures of a known biomarker derived from SAHA-treated cell lines and PBMCs of SAHA-treated patients.

**Results:**

We found cDNA hybridization targets detect at least 20 times more specific differentially expressed signatures (2597) between 1% liver and 1% brain in globin-supplemented samples than the PNA (117) or no treatment (97) method at FDR = 10% and p-value < 3x10-3. In addition, we found that the *ex vivo *derived gene expression profile was highly concordant with that of the previously identified SAHA pharmacodynamic biomarkers.

**Conclusions:**

We conclude that an amplification method for gene expression profiling employing cDNA targets effectively mitigates the negative impact on data of abundant globin transcripts and greatly improves the ability to identify relevant gene expression based pharmacodynamic biomarkers from whole blood.

## Background

Whole blood is a complex mixture of cell types that are exquisitely acute sensors of the body's physiological state [1-8]. It has long been the source tissue used in numerous tests for the identification of disease and the monitoring of disease progression. Peripheral blood is easily accessed and the available analytical techniques are well-established with a focus on the quantification of various chemical analytes (proteins, lipids, etc). Yet, gene expression profiling of peripheral whole blood has yet to be employed broadly. With the proliferation of whole genome analysis techniques, and their potential utility as both prognostic and diagnostic tools, there is a growing need to utilize readily available peripheral blood for techniques such as SNP analysis, copy number variation analysis and genome-wide gene expression.

Even though peripheral whole blood is one of the most easily accessed tissues for whole genome gene expression profiling, there are a number of technical challenges. The first is mRNA stabilization and isolation. The introduction of point-of-collection products that stabilize nucleic acids for whole blood (i.e. PAXgene, Tempus) has proven to be a major advance in the reduction of process-related artifacts [9,10]. These systems generally allow the collection of whole blood directly into a stabilizing reagent that prevents further RNA transcription and degradation. Although these stabilization technologies are readily available, many studies employ methods subject to sample storage or processing artifacts [11]. For example, it has been shown that delays in processing blood samples can lead to changes in expression of thousands of genes [9,12,13].

Another challenge is that the specificity and sensitivity of a given RNA profiling platform are affected by the abundance and variability of the globin transcripts, which can comprise up to 70% of mRNA in a whole blood extract [14]. In a basic research setting (as opposed to a clinical setting), scientists have circumvented the reticulocyte problem by isolating peripheral blood mononuclear cells (PBMCs) However, isolating PBMCs is difficult for many clinical sites to achieve and inadvertent delays in processing time can lead to processing biases that can reduce discovery power of expression profiles [12]. To improve the laboratory assays and increase discovery power, several commercially available solutions have been developed to reduce or mitigate the effects of excess globin transcripts on microarray hybridization signal. These can be classified into two strategies. The first approach focuses on minimizing the amplification of globin specific messages in amplified cRNA. These methods include physically removing globin transcripts from total RNA by hybridization to anti-globin oligonucleotides affixed to magnetic beads (GLOBINclear™, [15]) or by blocking the amplification of globin transcripts using oligonucleotides of nucleic acid analogs (PNA, LNA), which when bound to a transcript prevents its amplification by reverse transcriptase [16]. The PNA approach has been recommended by Affymetrix [17]. Because of sample manipulation, GLOBINclear has the potential to adversely affect the integrity of total RNA [18], is difficult to scale up and requires species-specific reagents (Wright, unpublished observations). Since we had evaluated this method previously, it was not included in this study. The PNA-based technique is simple and scalable, but PNA design is difficult and costly to expand for other species. Both techniques generate a hybridization target composed of cRNA and rely on the post-RNA isolation manipulation of the samples prior to or at the first step of mRNA amplification, leading to potential processing bias in gene expression data.

A second approach does not specifically restrict amplification of globin transcripts; rather it relies on the high specificity of DNA-based hybridization [19,20]. In these methods, all transcripts, including globin, are amplified to produce complementary cDNA. It is believed the high specificity of DNA-DNA interactions reduces cross hybridization signal due to excess globin, thereby reducing artifactual signals. The specific technology used in this manuscript is NuGEN's Ribo-SPIA, a highly sensitive method for generating cDNA target from nanogram quantities of total RNA. The methodology amplifies target mRNA using a novel template generation and isothermal strand displacement strategy [19,21]. It has recently been improved with the addition of the Whole Blood reagent (WB) that optimizes the amplification for whole blood samples.

Many of the current evaluations of globin mitigation strategies are based on biological models in which ground truth is largely unknown. Therefore, conclusions are based on semi-quantitative analysis of present calls [22] or on a lack of technical replicates [18]. In another study, differential expression was not detected in whole blood processing protocols, including two mitigation protocols [23]. Even though the above studies qualitatively show that mitigation approaches have the potential to improve sensitivity and specificity, there are remaining questions of globin impact on power to discover relevant biological signals from gene expression profiling of whole blood.

In order to identify an optimal strategy for the identification of pharmacodynamic biomarkers in whole blood, we established two model systems to identify and apply the best technique. First, we used a progressive globin transcript spike-in strategy to compare three methods to process samples, including two leading globin mitigation methods. Biological differences are modeled by spiking 1% liver or 1% brain total RNA. Jurkat RNA was used as a background for globin transcript spike-in to estimate potential bias in background. Identical sets of spiked-in samples were profiled at two different labs to check the reproducibility of the results. Then, we applied the more sensitive technique to a model system in which whole blood was treated *ex vivo *with a pharmacological agent to mimic a compound pharmacodynamic biomarker. To determine whether the drug-induced expression patterns observed were biologically meaningful, these data were then compared to a published pharmacodynamic biomarker derived from compound-treated cell lines and from peripheral blood mononuclear cells (PBMCs) isolated from patients treated with the compound in a Phase Ib clinical trial [24].

## Methods

### Identification of an Optimized Globin Mitigation Strategy

Unless noted, the generation of samples has been described previously [14]. The sample set used in this study is summarized in additional file 1. Variability in the levels of globin transcripts in a sample was modeled by spiking the baseline sample with 0%, 2%, 4% or 8% (by mass in total RNA) of synthetic globin message (a 3:1 mixture of alpha and beta globin, see the above reference for a complete description). This range of globin supplementation was chosen to mimic a wide range of potential globin levels. As noted by Wright et al., both the range and variability of globin levels that contribute to a globin-interference artefact [14]. To simulate differential expression, samples were spiked with 1% of Brain or 1% Liver (w/w) total RNA into Jurkat total RNA. This spiking strategy (with globin, brain and liver RNAs) was also applied to a pool of PAXgene-collected whole human blood from volunteer donors, and similar data were obtained (data not shown).

### RNA samples

Jurkat, brain and liver total RNAs were purchased from Ambion (Foster City, CA). Globin transcripts (a mixture of alpha and beta) were synthesized as previously described [14]. Samples were quantitated by UV spectrophotometry and quality was assessed using an Agilent Bioanalyzer and the Agilent RNA 6000 Nano kit (data not shown).

### Gene expression profiling

Aliquots of each sample were profiled for gene expression with or without globin mitigation using an automated version of the Affymetrix reverse transcription-*in vitro *transcription protocol (RT-IVT) as described by the manufacturer (Affymetrix Inc., Santa Clara, CA). PNAs were designed as described by Affymetrix [17] and purchased from PanaGene (Daejeon, South Korea). Samples were treated with the PNA cocktail as described and profiled using the same RT-IVT protocol as the control. A third aliquot of each total RNA was amplified using the NuGEN Ovation Whole Blood Solution protocol (NuGEN, Inc., San Carlos, CA) as described by the manufacturer [25]. Amplified biotin-labeled material was hybridized to custom-designed Affymetrix microarrays (GEO accession GPL6793), one sample per array. Hybridization, washing and scanning were completed as recommended by the manufacturer.

### Ex vivo human whole blood studies

300 mL of whole blood from 10 anonymous and consenting adults (5 male and 5 female) was collected into a blood collection bag with citrate dextrose phosphate adenine (CDPA) (Terumo Medical Corp, Somerset, NJ). The blood samples used as the basis for the procedures described in this manuscript were drawn from healthy volunteers for development of novel laboratory techniques, thus the provisions of the Declaration of Helsinki are not applicable. Each volunteer donor read and signed an informed consent document that described the potential risks involved with giving a blood sample through venipuncture. The blood samples were drawn by a certified phlebotomist. 25 mL of each donor's blood was then aliquoted into 3 different canted neck 75 cm^2 ^culture flasks (Corning, Corning NY). One aliquot of whole blood received DMSO as a vehicle control; the other two aliquots were treated with Suberoylanilide Hydroxamic Acid (SAHA) to a final concentration of either 0.33 μM or 3.3 μM. The culture flasks were incubated at 37°C with 5% CO_2_. At 0, 3, 6 and 12 hours multiple 2.5 mL samples were drawn from each of the flasks and immediately mixed with PAXgene RNA stabilization reagent. Time points and doses were chosen in order to maximize the likelihood of detecting a SAHA induced change in mRNA profiles. Samples were stored at -80°C. Total RNA was extracted from the 0, 3, and 6 hour samples using a custom semi-automated version of the vendor's PAXgene 96 Blood RNA system. RNA Quality was assessed as described above, and prepared for microarray array hybridization using a semi-automated version of the NuGEN Ovation WB protocol with biotin labelling [25]. Samples were hybridized to Rosetta custom Affymetrix GeneChip arrays (see above) following the vendor's recommended protocols.

### Data processing and analysis

Microarray data quality was assessed using standard metrics [26]. RMA was used for data normalization and processing [27]. Analysis was done using log_2 _scale intensity values. Genes significantly (p-value < 0.01, abs(rho) > 0.6) correlated to the amount of spiked-in globin were defined as globin artifact. Correlation does not measure the amplitude of the globin artifact or the amount of noise it introduces. We have chosen the standard deviation of expression values rather than covariance to quantify the amplitude of the genes correlated to spiked globin due to a simpler implementation and associations with effect size measured by Cohen's distance. Data was analyzed using Matlab, Spotfire DecisionSite, SAS and R. A t-test was performed to detect significant differences between liver and brain spiked-in samples. The p-value threshold for this test used to declare a significant differential expression value between liver and brain spiked samples was set such that the false discovery rate (FDR) was constrained to be < 0.1, as determined by permutation [28,29].

ROC analysis was done as follows: the true positive rate was estimated using p-value of t-test between liver and brain spike-in samples; false positive rate was estimated using t-test after permutation of sample indexes. Permutation is constrained so that each group has equal number of liver and brain spiked samples. This will ensure that false positive rate is not inflated by biological differences.

## Results and Discussion

### Globin mitigation improves microarray data quality

In order to quantify the impact of excess globin on hybridization quality, we developed a controlled system using Jurkat RNA spiked with varying levels of globin transcript as well as low levels (1%) of brain and liver RNA supplements. This synthetic system provides an objective means of identifying signals related to globin abundance versus those of other sources of biological variability. Brain and liver spike-ins yield a well-defined differential gene expression pattern, which can be used for quantifying the impact of globin on signature gene detection. Previous work in our laboratory and by others has demonstrated that excessive levels of globin transcripts can induce a data artifact through promiscuous cross-hybridization to microarray probes [14,22]. Consistent with this, both Scale Factor (a measure inversely proportional to array intensity) and Percent Present (a measure of discrimination between probes and background) are negatively impacted by increasing amounts of globin. PNA treatment was found to improve the Percent Present metric by approximately 10 percent, while the cDNA amplification improved this metric by 25 percent and reduced the background correlated to the amount of globin spiked into each sample (additional file 2). Although hybridization quality is an important metric, it is not always directly related to biological signal.

**Figure 1:**
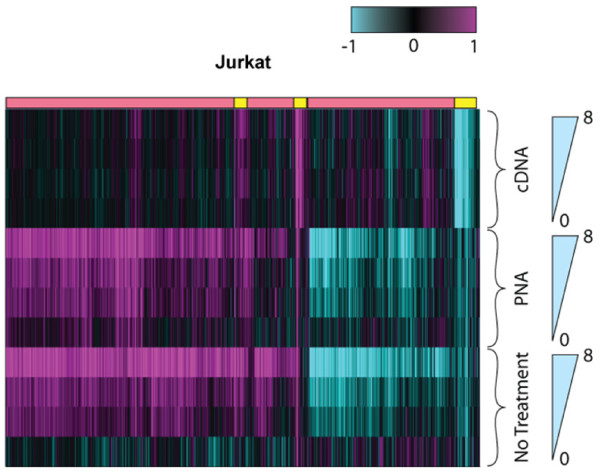
**Gene clustering of all signatures associated with globin cross-hybridization and tissue specific effects**. The x-axis corresponds to clustered genes. Rows correspond to samples. Jurkat RNA samples spiked with brain were referenced to a sample with spiked liver and no globin within each treatment. Samples are sorted by the amount of spike globin. Amount of globin is indicated by triangles and ranged from 0% to 8%. Yellow and magenta bars indicate tissue specific effect and globin artifact, respectively. Data are on a log2 scale.

Figure 1 depicts a heat map with the experiments grouped first by mitigation technology, then by the amount of globin spike-in. Expression ratios between brain and liver containing samples were derived within a given globin concentration and mitigation strategy to account for differences in protocol-associated intensity. Genes correlated to the amount of spiked-in globin transcript and demonstrated tissue specific expression (p-value < 0.003 and FDR = 10%) were clustered using hierarchical clustering. Note that in this controlled system, the vast majority of genes in the signatures derived from both the PNA and no treatment control are correlated to globin content rather than of genes differentially regulated between brain and liver (data not shown). Greater than 23,000 transcripts correlated significantly (p-value < 0.01, abs(rho) > 0.83) to the amount of globin transcript spiked into each sample across all arrays (figure 1, red bars). Only the cDNA protocol mitigates the globin artifact in a robust enough manner to reveal the smaller underlying Brain/Liver signature (figure 1, yellow bars).

These results support the hypothesis that globin-related cross-hybridization is the main source of the artifact. Reducing globin cross-hybridization by either SPIA amplification of samples or PNA blockage of reverse transcription improves average probe intensity and discrimination from background. Therefore, correlation between the amount of globin and gene expression signal is a robust metric for measuring globin interference.

**Figure 2:**
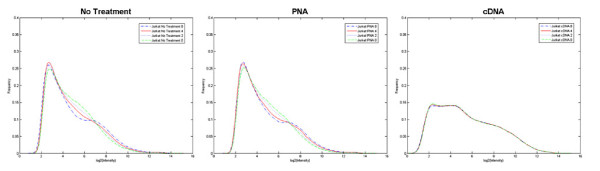
**Globin affects global changes in density distribution of intensity**. RMA-derived intensity values were binned and plotted against their frequency for Jurkat samples spiked with 4 different concentrations of globin (0, 2, 4, 8%).

Analysis of the distribution of microarray intensities for each method also reveals significant differences between the technologies. Figure 2 plots the density distribution of probeset intensities for both mitigation technologies and processing without globin mitigation. These plots show a shift in density distribution for the cDNA target samples, and very little difference between the PNA method and no treatment control. Increasing globin transcript abundance results in a progressive downshift of signal density between log2(Intensity) of 4 and 8 for the PNA and no treatment controls. Given that most of the probesets fall within this intensity range, the impact of globin abundance will have a global effect on array performance. The change in shape of the density distribution will result in normalization artifacts as well, since the majority of normalization techniques assume intensity distributions are similar between related samples. The cDNA target distribution shows no shifts due to globin abundance. In addition, cDNA targets exhibit more uniform detection and discrimination of low-expression genes by increasing expression signal across a wider range of low-intensity probes. Another important characteristic of cDNA targets is the reduction of background intensity, which is represented by the shift in the peak maxima. Peak maxima typically reflect the background intensity on the array. The intensity distribution of cDNA targets is not sensitive to globin content and showed greater discrimination between low-expression genes and background, which is indicated by two maxima.

### cDNA amplification significantly reduces the number of genes correlated to globin

**Figure 3:**
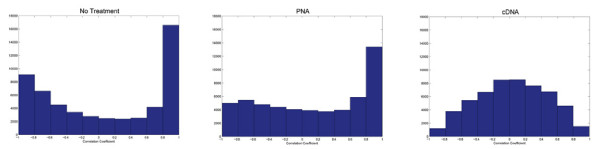
**Distribution of Pearson Correlation coefficients between spiked in globin and gene expression of Jurkat samples**. The calculated amount of globin in each Jurkat sample was correlated to the expression of all genes for each treatment (p-value < 0.01; abs(Rho) = 0.83). The Pearson Correlation coefficient values were binned and plotted against frequency. For the No Treatment, PNA and cDNA treatments, the number of genes significantly correlated to globin was 23290, 15912 and 1799, respectively. The significance threshold for correlation is set at p < 0.01, which corresponds to a magnitude of correlation coefficient of more than 0.83.

Genes whose expression increases in proportion to the amount of globin added to the sample can readily be identified as globin-induced artifactual discoveries. In order to quantify the effect of globin interference on gene expression data, we calculated the Pearson correlation coefficient between expression levels and globin abundance for each gene. Figure 3 shows the frequency distribution of correlation coefficients for each treatment. The large number of positively or negatively correlated probesets could be explained as a result of RMA compensating for normalization of the highly correlated genes and imbalance in mRNA content. PNA treatment reduces the number of genes significantly correlated (p < 0.01) to globin from 23,290 in no treatment control to 15,912 genes (table 1). The distribution of correlation coefficients for cDNA targets is almost normal, which is the expected result of strong mitigation, with just 1,799 genes significantly correlated to globin transcript abundance and no strong normalization artifact apparent (table 1 and figure 3).

**Table 1:** Quantitative assessment of globin interference and tissue-specific signatures.

	Probesets Correlated to globin (p-value < 0.01)	Tissue specific probesets FDR = 10% ** (critical p-value)	Standard Deviation of intensity for globin-correlated genes	Power ^++^
No Treatment	23290	97 (2e-4)	0.36	11%

PNA	15912	117 (2e-4)	0.30	18%

cDNA	1799	2597 (3e-3)	0.12	90%

Globin Related genes are those that have a significant correlation in expression magnitude to the amount of globin in each sample. Tissue Specific genes are those that are associated to a 1% brain vs. 1% liver expression pattern. Standard Deviation refers to the variability in globin interference genes correlated to the variation in globin content.

** Critical p-value is in parenthesis

++ Power to detect 1.4 fold change at p-value = 0.01, 4 samples per group

### cDNA amplification significantly improves gene expression discovery power

To determine the impact of globin transcript mitigation on discovery power, we calculated statistical power by using the SAS power procedure. Both the PNA and cDNA strategies improved data by reducing the amount of detectable globin interference. PNA treatment decreased interference by ~30%, as measured by the number of genes correlated to globin with PNA treatment compared to the no-treatment control (figure 3 and table 1), while cDNA hybridization reduced globin-induced noise by more than 90%. First, genes differentially expressed (tissue-specific genes) between 1% liver and 1% brain spiked samples were detected using a t-test. The critical p-value was set to control false discovery rate (FDR) at 10% for each processing method. FDR was determined using a permutation approach (see Methods). The no-treatment, PNA, and cDNA critical p-values were set equal to 4e-4, 4e-4 and 3e-3 respectively. We observed higher FDR for samples processed using PNA or no treatment at the same p-values compared to the cDNA samples. In order to keep FDR = 10%, we had to reduce the critical p-value cut off for the analysis of PNA and no treatment samples. The number of significant genes differentially regulated between 1% liver and 1% brain is equal to 97 for no treatment, 117 for PNA and 2,597 for cDNA. The statistical power of the detected changes is more than 90% at p-value of 1e-4.

**Figure 4:**
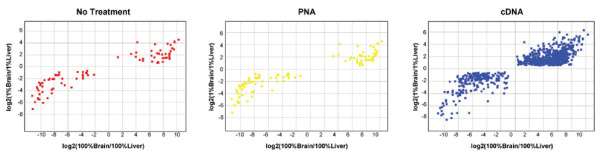
**Correlation of 1% brain/liver signatures to 100% brain/liver signatures**. Ratios for differential gene expression in brain/liver samples were calculated and plotted against each other for 1% brain/liver and 8% globin in Jurkat RNA versus 100% brain/liver RNA.

As a further validation of the approach, significant changes in gene expression of globin-spiked samples were plotted against related changes in 100% brain vs. 100% liver. The correlation of signature genes shown in figure 4 confirms that detected changes are representative of biological differences between liver and brain.

Another way to evaluate effects of mitigation is to estimate the statistical power necessary to detect differential regulation under given experimental conditions. Variation in expression data was estimated using mean standard deviation of intensity on a logarithmic scale for genes significantly correlated to globin. This estimate was used because nearly 50% of probesets significantly correlate to globin addition. The standard deviations were 0.36 for no treatment, 0.3 for PNA and 0.12 for cDNA (table 1). cDNA hybridization allowed for the detection of 1.4-fold change in expression at p ≤ 0.01 with 90% power, assuming 4 samples per group. PNA and no treatment power are 18% and 11%, respectively, under the same conditions (table 1). In order to compensate for loss in statistical power in PNA and no treatment samples, the number of samples per group needs to be increased from 4 to 9. Thus, this shows that while the loss in sensitivity is not fatal to biomarker discovery, more sample replicates are required to achieve the same statistical power. While both globin mitigation strategies increase the number of genes identified as differentially-expressed between brain and liver, the cDNA methodology substantially increases the number of genes detected relative to both the control and PNA methods.

**Figure 5:**
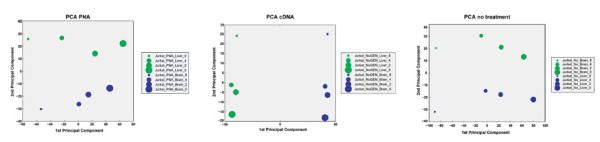
**Principal Component Analysis of tissue-specific and globin-related gene expression**. PCA was performed on the expression values of Jurkat samples supplemented with 1% brain or liver RNA. The circles indicate the amount of globin while the color indicates whether the sample was spiked with brain or liver.

We performed a Principal Component Analysis (PCA, figure 5) of the data derived from differential brain versus liver signatures in order to identify and quantify the sources of variation in the data. Plotting the values for the first two principal components shows a clear difference between the cDNA methodology and the other two protocols. For both the PNA and no treatment conditions, the first principal component is driven by the amount of globe spiked in, contributing to 70% of the total variation in those samples. The second principal component (10% of the variation) was the brain/liver signature. However, the first principal component of the cDNA target data is driven by the brain/liver signature, detected following globin mitigation. The second principal component was the amount of globin in the samples. This analysis provides a quantitative demonstration that there is very little difference between PNA and no treatment and that these conditions were essentially unable to significantly resolve a signature between samples spiked with brain or liver RNA.

**Figure 6:**
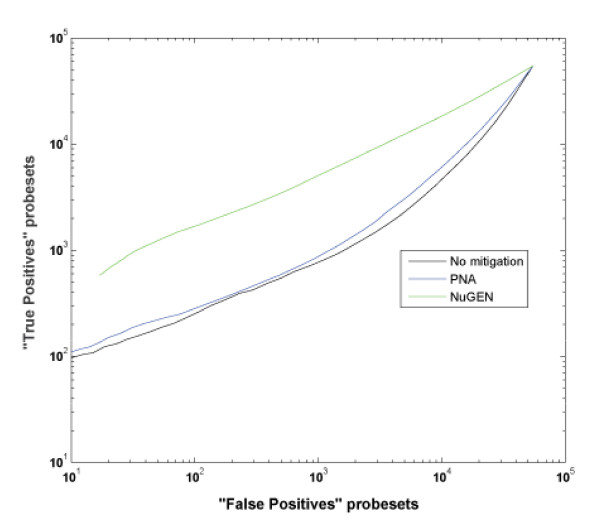
**Receiver Operator Characteristic curves of 1% brain vs 1% liver signature detection**. The total number of genes detected by a t-test at a specific p-value are plotted against the number of false positive genes detected at the same p-value. False positives were calculated using permutations controlled for equal number of liver and brain samples in each group. ROC analysis was done as follows: the true positive rate was estimated using a t-test between liver and brain spike-in samples; false positive rate was estimated using t-test after permutation of sample indexes. Permutation is constrained so that each group has equal number of liver and brain spiked samples. This will ensure that false positive rate is not inflated by biological differences.

A Receiver Operator Characteristic curve plot is used to evaluate discrimination power between different platforms [30]. It is also a means of visualizing the relationship between sensitivity and specificity where the abscissa indicates the number of false positive genes detected by t-test between two groups with no biological differences and the ordinate is the total number of genes detected in the Jurkat samples spiked with either liver or brain total RNA. The Ribo-SPIA method detects a far greater number of significant genes at any level of "false positive" detection selected (figure 6).

### Demonstration that cDNA Target Reveals a Physiologically Relevant Expression Profile in Whole Blood

To demonstrate that cDNA targets were able to reveal a meaningful biological gene expression signature in whole blood, we developed an *ex vivo *platform for putative biomarker identification (see Methods for details). Whole blood collected from consenting, healthy volunteers was dosed with two different concentrations of Suberoylanilide Hydroxamic Acid (SAHA), a histone deacetylase inhibitor used in cancer treatment or vehicle (dimethylsulfoxide). Sample aliquots were removed at two different time points and mixed with PAXgene reagent to stabilize the transcriptional profile prior to RNA extraction and analysis on Affymetrix microarrays.

We designed this experiment to identify gene signatures that were regulated in both a time-and SAHA dose-dependent manner. By definition, these genes would be potential markers of SAHA pharmacodynamic effects in whole blood. We expected that these gene sets would have significant overlap with published SAHA response data sets from lymphoctyes of SAHA-treated patients or treated lymphocyte cell lines [31]. Additionally, it is reasonable to assume that this experimental design would also identify genes related to perturbations of whole blood not easily identified in other model systems. Table 2 shows an analysis of the intensity data for genes that were significantly regulated by time and dose. Even at restrictive p-values (< 0.001) almost 5,000 genes can be identified. The identification of a time-dose regulated set of genes provides confidence that the experimental design successfully modelled a drug induced signature.

**Table 2:** ANOVA analysis of time and SAHA dose dependent genes.

Regulation	Number of transcripts (p < 0.01)	Number of transcripts (p < 0.001)
Down	3936	2764

Up	3814	2240

**Figure 7:**
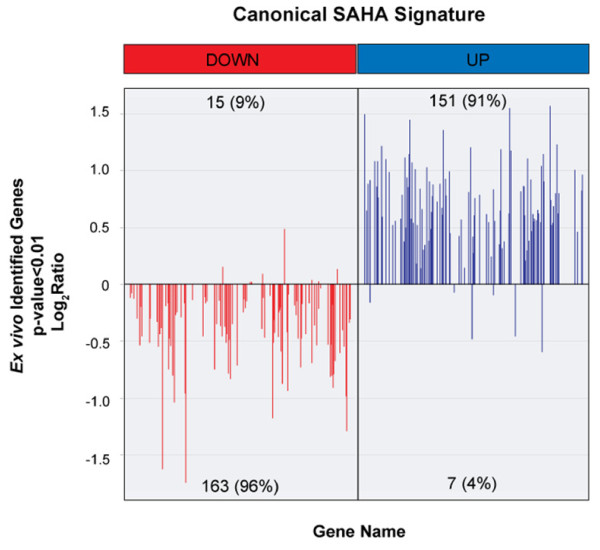
**Concordance between canonical SAHA signature and the signature identified by the ex vivo profiling method**. 50% of the canonical SAHA signature (336 genes) is represented on the custom Affymetrix GeneChip array, with a p < 0.01. More than 90% of the genes are regulated in the same direction between the two datasets.

To confirm that the significantly regulated genes reflect changes in pathways known to be impacted by histone deacylases such as SAHA, we compared the Ribo-SPIA-identified genes to the canonical SAHA response signature [24]. This signature was derived from a number of data sets and was shown to be consistently regulated in different tissues, cell lines and in a previous Phase Ib *in vivo *blood study [24,31]. Concordance between the canonical signature and the *ex vivo *signature was assessed by analyzing the performance of the *ex vivo *signature on the probe sets best matched to the canonical signature. Down-and up-regulated canonical SAHA signature genes are represented on the custom Affymetrix microarray by 324 probe sets and 333 probe sets, respectively. Concordance of detected regulation is presented in figure 7. Approximately 85% of genes show similar regulation between the canonical and *ex vivo *gene lists without statistical cuts (data not shown). 336 (50%) genes of the canonical SAHA gene list were significantly changed in the *ex vivo *experiment with more than 90% concordance in the direction of regulation (p << 0.01 Fisher exact test). These included a number of genes previously identified as SAHA response genes in the PBMCs of treated patients, which included the down regulation of *MYC *and up regulation of *GADD45B *[24].

## Conclusions

Blood is a critical tissue for the understanding of disease and the development of disease treatments. It is a ubiquitous tissue that interacts throughout the body and literally acts as a sensor of physiological conditions [1,2]. While many assays exist to extract this critical knowledge from blood for proteins, lipids and single genes, development of genome-based biomarker assays has been a challenge. This is due to the high and variable levels of globin transcripts that interfere with achieving significant sensitivity [14]. To this end, several commercial solutions have been developed to prevent the generation of globin transcripts during sample preparation. We and others have shown that many of these methods do improve data quality (figure 1; [14,18,22]). However, using Ribo-SPIA amplification, we have demonstrated that the globin transcript can be fully represented in the target and its effect on hybridization data can be ameliorated through the highly-specific properties of DNA:DNA binding.

Most microarray platforms typically utilize a fixed probe length of DNA, whether spotted in place or synthesized *in situ*. This critical fact defines much of the performance of the microarray in terms of sensitivity (DNA will allow a certain amount of promiscuous cross hybridization effecting background determination) and specificity (the fidelity of hybridization between the probe and the target). Standard amplification techniques rely on the RT/IVT method developed by Eberwine and Van Gelder [32]. This method amplifies mRNA and incorporates the necessary label using an *in vitro *transcription step that is robust and efficient. The amplified material produced is a cRNA whose characteristics for sensitivity and specificity are acceptable, but not as good as a DNA target. DNA has been shown to perform better as a hybridization target than RNA, since it is highly specific and less susceptible to cross-hybridization [20]. These characteristics also support the use of DNA as a means of mitigating the effects of globin, and potentially other highly abundant interfering transcripts.

Evaluation of the hybridization characteristics show that cDNA probes generated by Ribo-SPIA amplification perform better than using the standard cRNA method of amplification and labeling. cDNA hybridizations have greater intensity (low Scale Factor) and better discrimination between true signal and background (measured as a higher percentage of present calls) (additional file 2). Not only are there improvements in hybridization metrics, but the deleterious effects of globin cross-hybridization are reduced. As seen in figures 1 and 5, and quantified in table 1, the correlation between the amount of globin in a sample and the number of false positive signatures is greatly reduced when either globin mitigation strategy is used. However, we found that the Ribo-SPIA method significantly outperformed the PNA method. Indeed, there is an improved detection sensitivity of nearly 4-fold, a reduction of the globin artifact by 5-fold and an increase in statistical power (signal to noise) of more than 3-fold. The loss of correlation between the amount of globin in the sample and the number of false detections indicates the benefits of this approach. This improved performance was consistent whether the background sample was of either a cell line or whole blood origin.

Concomitant with a reduced correlation between globin and false positive signatures is an increase in the number of true signatures detected. Irrespective of globin interference, it is useful to measure the sensitivity of all methods. When comparing the spiked-in liver vs brain signatures, the Ribo-SPIA protocol identified 4,000 more significant genes than the standard no treatment control. PNA had little to no effect in sensitivity with an increase of less than 200 genes (table 1). This benefit is magnified in the presence of cross-contaminating globin. Figures 2 and 5 show the benefits of globin mitigation. Both the Ribo-SPIA and PNA methods increase the number of true detections (as measured by the number of brain or liver signatures detected) when compared to no treatment. As before, the Ribo-SPIA protocol is far superior to the standard PNA protocol. Figure 6 shows a ROC-like analysis where genes associated with globin amount are considered false positives and the total number of signatures detected is derived by building a ratio between the Jurkat spiked with brain and Jurkat spiked with liver. This presentation shows that for a given level of false positives attributable to globin cross-hybridization, both the Ribo-SPIA and PNA protocols are more sensitive than a no treatment control, with the Ribo-SPIA significantly outperforming PNA.

During the preparation of this manuscript, a number of other teams have published studies evaluating methodologies for whole genome expression profiling from whole blood. Many of these used similar methodologies for objectively measuring expression profiling performance [14,22]. Others have noted the benefits of usincDNA targets for profiling, although in some cases it was noted that earlier versions of the Ribo-SPIA protocol were used [18]. It should be noted that we used the NuGEN Ovation WB kit, which is an improved method over early versions.

Other recent work has compared profiling using cDNA to other methods, including direct isolation of PBMCs [23]. In any research, the cause of negative results is often unknown and dismissed based on several reasons. For example, it was reported that robust transcriptional signature of acute graft rejection in tissue biopsies could not be detected in whole blood even after using cDNA-based amplification and hybridization [23]. The cause is unknown and could be due to the biological relevance of whole blood in detection of graft rejection or inability to fully mitigate globin effects.

There are several examples in the literature of *ex vivo *gene expression profiling as well as experiments looking at the SAHA-induced expression profiling [31,33-37]. The latter generally rely on the isolation of PBMCs in order to mitigate globin contamination. This extra processing can induce signatures of its own and thus reduce sensitivity [10,12,38,39]. A significant benefit of the NuGEN Ovation WB protocol is that such extra manipulation is not necessary and pre-amplification noise is not introduced. The goal of the study was to demonstrate the utility of cDNA targets for whole blood gene profiling. Using a cDNA target derived from the Ribo-SPIA protocol, the number of genes correlated to globin input was reduced by 5-fold compared to a no treatment control, with a 4-fold increase in tissue-specific genes. Although the study was not specifically designed or powered to identify new clinically-relevant biomarkers, it was designed to capture the time-and dose-dependent biological response of whole blood to SAHA administration. These data support the concept that cDNA hybridization to microarrays is a valuable methodology for identifying clinically-relevant gene expression patterns in whole blood and reveal previously obscured biomarkers.

## Competing interests

All authors were employed by Merck & Co. at the time the work was completed. The authors have no other competing interests to declare.

## Authors' contributions

MP conceived of the study design, participated in the *ex vivo *dosing study, and led the drafting and editing the manuscript. CW contributed to the study design, developed the spike-in samples, and participated in the *ex vivo *dosing study. YR contributed to the study design, participated in the expression profiling assays and participated in the *ex vivo *dosing study. DA participated in the expression profiling assays and participated in the *ex vivo *dosing study. HC completed all of the extraction of total RNA from blood samples. BL provided project and sample management support. AL and MN completed the analysis of the SAHA data. MM assisted with the data analysis and participated in drafting and editing of the manuscript. SL completed the analysis of the protocol selection study, participated in the analysis of the SAHA data and participated in the drafting and editing of the manuscript. All authors read and approved the final manuscript.

## Supplementary Material

Additional file 1 **Sample set used for globin spike-in experiments**. Jurkat RNA samples were supplemented with a physiologically-relevant range of globin mRNA. See Wright et al, for a complete description [14]. Click here for file

Additional file 2 **Hybridization quality assessment**. Scatter plot of scale factor values versus percent of present calls. Percent of present calls is the percent of probesets with a significant difference in intensity between perfect match (PM) and mismatch (MM) probes. Scale factor is inversely proportional to the array intensity. Colors indicate protocol and the size of squares corresponds to the amount of spiked globin (see additional file 1). Each data point corresponds to an array. Click here for file
