# Supplementary material for: cDNA targets improve whole blood gene expression profiling and enhance detection of pharmocodynamic biomarkers: a quantitative platform analysis
Source: J Transl Med. 2010 Sep 25;8:87. doi: 10.1186/1479-5876-8-87 (PMC2954848; doi:10.1186/1479-5876-8-87)
Supplement: Additional file 1 — Sample set used for globin spike-in experiments. Jurkat RNA samples were supplemented with a physiologically-relevant range of globin mRNA. See Wright et al, for a complete description [14]. [file 1479-5876-8-87-S1.DOC]

Additional File 1

| Sample | Baseline | % of Spiked Globin | Spiked Tissue RNA |
| --- | --- | --- | --- |
| 1 | Jurkat | 0 | 1% Liver |
| 2 | Jurkat | 2 | 1% Liver |
| 3 | Jurkat | 4 | 1% Liver |
| 4 | Jurkat | 8 | 1% Liver |
| 5 | Jurkat | 0 | 1% Brain |
| 6 | Jurkat | 2 | 1% Brain |
| 7 | Jurkat | 4 | 1% Brain |
| 8 | Jurkat | 8 | 1% Brain |
