# Supplementary figures and images for: cDNA targets improve whole blood gene expression profiling and enhance detection of pharmocodynamic biomarkers: a quantitative platform analysis
Source: J Transl Med. 2010 Sep 25;8:87. doi: 10.1186/1479-5876-8-87 (PMC2954848; doi:10.1186/1479-5876-8-87)

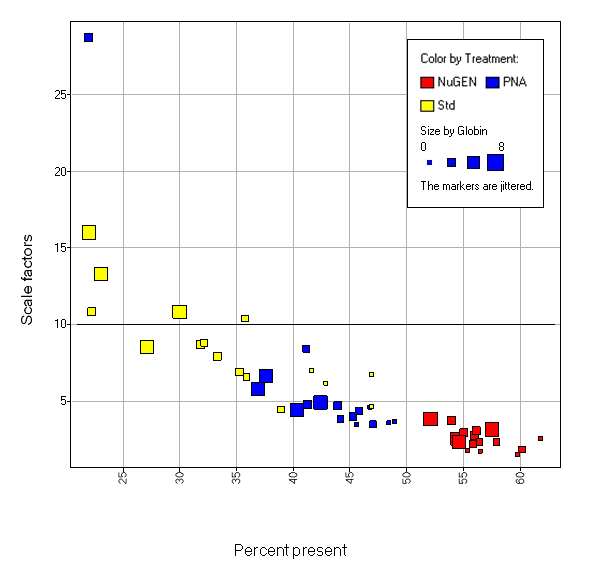

Supplement: Additional file 2 — Hybridization quality assessment. Scatter plot of scale factor values versus percent of present calls. Percent of present calls is the percent of probesets with a significant difference in intensity between perfect match (PM) and mismatch (MM) probes. Scale factor is inversely proportional to the array intensity. Colors indicate protocol and the size of squares corresponds to the amount of spiked globin (see additional file 1). Each data point corresponds to an array. [file 1479-5876-8-87-S2.TIFF]
